# Lessons Learned for Pathogenesis, Immunology, and Disease of Erythrocytic Parasites: *Plasmodium* and *Babesia*

**DOI:** 10.3389/fcimb.2021.685239

**Published:** 2021-08-03

**Authors:** Vitomir Djokic, Sandra C. Rocha, Nikhat Parveen

**Affiliations:** ^1^Department for Bacterial Zoonozes, Laboratory for Animal Health, French Agency for Food, Environmental and Occupational Health & Safety, UPEC, University Paris-Est, Maisons-Alfort, France; ^2^Department of Microbiology, Biochemistry and Molecular Genetics, Rutgers New Jersey Medical School, Newark, NJ, United States

**Keywords:** *Plasmodium*, malaria, *Babesia*, babesiosis, pathogenesis, immune responses, vector borne protozoa, hemoparasite

## Abstract

Malaria caused by *Plasmodium* species and transmitted by *Anopheles* mosquitoes affects large human populations, while *Ixodes* ticks transmit *Babesia* species and cause babesiosis. Babesiosis in animals has been known as an economic drain, and human disease has also emerged as a serious healthcare problem in the last 20–30 years. There is limited literature available regarding pathogenesis, immunity, and disease caused by *Babesia* spp. with their genomes sequenced only in the last decade. Therefore, using previous studies on *Plasmodium* as the foundation, we have compared similarities and differences in the pathogenesis of *Babesia* and host immune responses. Sexual life cycles of these two hemoparasites in their respective vectors are quite similar. An adult *Anopheles* female can take blood meal several times in its life such that it can both acquire and transmit Plasmodia to hosts. Since each tick stage takes blood meal only once, transstadial horizontal transmission from larva to nymph or nymph to adult is essential for the release of *Babesia* into the host. The initiation of the asexual cycle of these parasites is different because *Plasmodium* sporozoites need to infect hepatocytes before egressed merozoites can infect erythrocytes, while *Babesia* sporozoites are known to enter the erythrocytic cycle directly. *Plasmodium* metabolism, as determined by its two- to threefold larger genome than different *Babesia*, is more complex. *Plasmodium* replication occurs in parasitophorous vacuole (PV) within the host cells, and a relatively large number of merozoites are released from each infected RBC after schizogony. The *Babesia* erythrocytic cycle lacks both PV and schizogony. Cytoadherence that allows the sequestration of Plasmodia, primarily *P. falciparum* in different organs facilitated by prominent adhesins, has not been documented for *Babesia* yet. Inflammatory immune responses contribute to the severity of malaria and babesiosis. Antibodies appear to play only a minor role in the resolution of these diseases; however, cellular and innate immunity are critical for the clearance of both pathogens. Inflammatory immune responses affect the severity of both diseases. Macrophages facilitate the resolution of both infections and also offer cross-protection against related protozoa. Although the immunosuppression of adaptive immune responses by these parasites does not seem to affect their own clearance, it significantly exacerbates diseases caused by coinfecting bacteria during coinfections.

## Introduction

Apicomplexan protozoa, *Plasmodium*, and *Babesia* species are described as erythrocyte-dwelling hemoparasites that cause serious morbidity in humans and animals alike (Allred, 1995; Springer et al., 2015), and are evolutionary-related organisms with overlapping life cycles, disease manifestations, and immune responses (Clark and Allison, 1974; Frolich et al., 2012). Out of 60 species, *P. falciparum, P. vivax, P. ovale, P. malariae*, *P. knowlesi, P. cynomolgi*, and *P. simium* infect humans to cause malaria (Milner, 2018; Garrido-Cardenas et al., 2019), while four others, *P. chabaudi, P. berghei, P. vinckei*, and *P. yoelii*, infect rodents (De Niz and Heussler, 2018). Similarly, out of over 100 *Babesia* spp. known, only *B. microti, B. duncani*, *B. divergens*, and *B. venatorum* are documented to infect humans in North America and Europe (Lobo et al., 2020), while others are identified more as infections of different animals (Lobo, 2005). Both protozoa are transmitted by vectors. *Anopheles* female mosquitoes transmit *Plasmodium*, and *Ixodes* species are vectors for *Babesia* transmission. While malaria is a very well-known disease, babesiosis has long been recognized as an economically important disease of cattle and other animals and has emerged as a reportable human disease in the United States only in 2011 (Lobo, 2005). Another major healthcare problem associated with babesiosis is that *Babesia* spp. can also be transmitted by blood transfusion; however, donated blood is usually not tested for this parasite. As a result, babesiosis is one of the most important pathogenic diseases transmitted by blood transfusion in the United States. It can also be transmitted vertically from mother to child during pregnancy (Wormser et al., 2015; Saetre et al., 2018) like *P. falciparum*. The proof for efficacy of blood screening was already proven some 40 years ago on *Plasmodium* spp., lowering its transmission greatly throughout the world. Screening of donated blood, once implemented for antibodies against, or DNA from *B. microti* showed association with a decrease in the risk of transfusion-transmitted babesiosis (Moritz et al., 2016). *P. falciparum* strain 3D7 genome of 22.8 Mb is distributed among 14 chromosomes ranging in size from approximately 0.643 to 3.29 Mb (Gardner et al., 2002). In comparison, *B. microti* possesses the smallest nuclear genome of 6.4 Mb among apicomplexan parasites with four chromosomes, thus limiting its metabolic functions (Cornillot et al., 2012), while the *B. divergens* genome size is ~10.7 Mb (Cuesta et al., 2014), indicating that more complex gene expression and regulatory systems are present in *Plasmodium* than *Babesia*.

**Figure 1:**
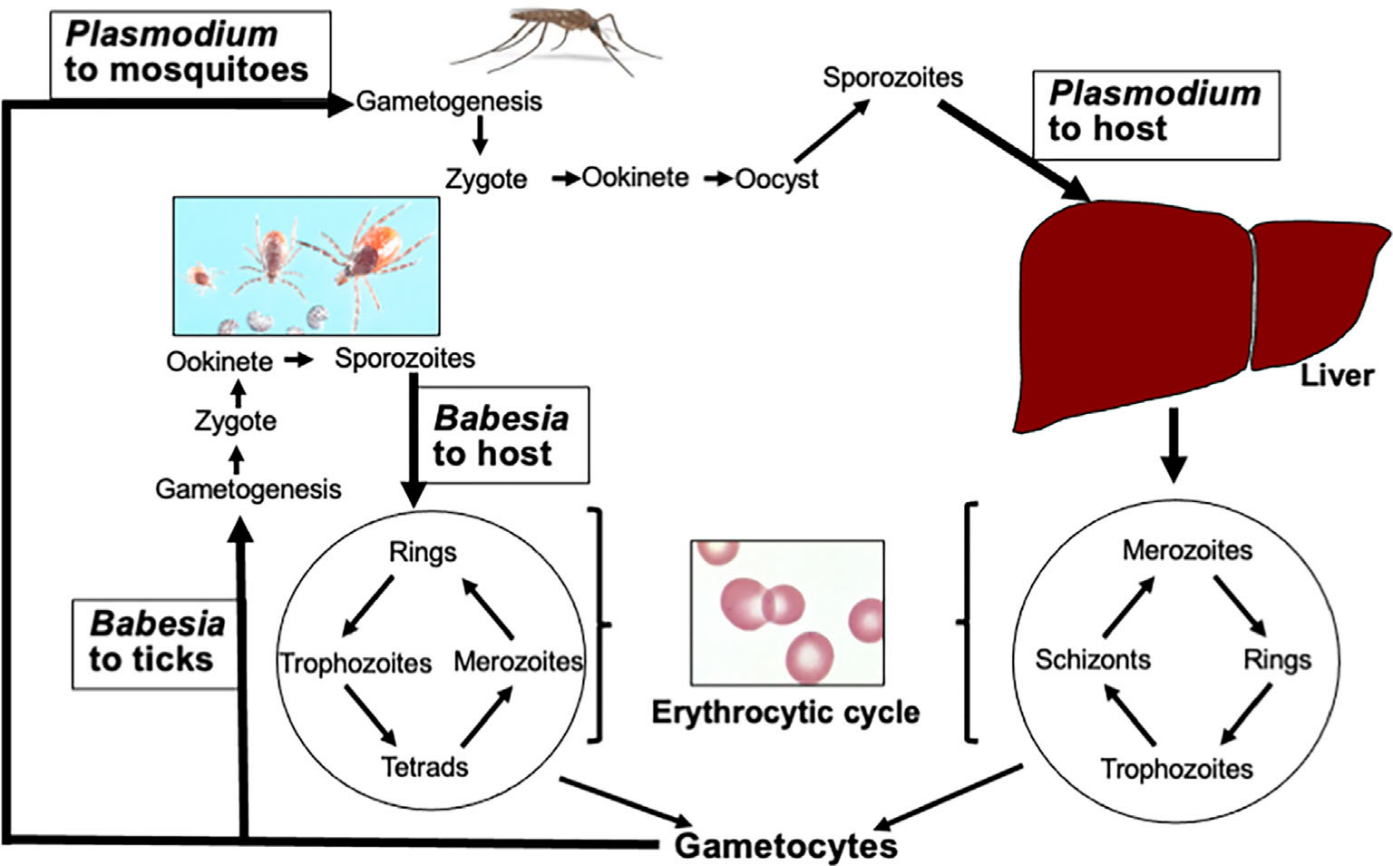
Life cycles of *Plasmodium* and *Babesia* species. After acquiring *Plasmodium* gametocytes from infected hosts, gametogenesis followed by fusion of gametes to produce zygote occurs in the midgut of Anopheles females. Ookinete passes through the midgut wall, and sporozoites are released from the oocyte into salivary glands that are transmitted to the host during blood meal. Similar steps are followed by *Babesia* species in ticks except it requires transstadial horizontal transmission of parasites from one stage of the tick developmental cycle to the next. After deposition in skin dermis, the haploid sporozoites of *Plasmodium* travel to the liver through blood and infect hepatocytes. After a single replication cycle, the merozoites released from hepatocytes enter the erythrocytic cycle. Invasion of hepatocytes is absent in the *Babesia* cycle. In addition, the *Babesia* cycle in RBCs differs from *Plasmodium* because unlike *Plasmodium*, it does not replicate in the parasitophorous vacuole, lacks PTEX secretion apparatus, and does not undergo schizogony. (Image of different stages of *Ixodes scapularis* ticks was generously provided by James Occi of Rutgers University at New Brunswick, NJ, USA.)

Transmissibility of *Babesia* species from animals to humans and vice versa allows through vector the involvement of wildlife as reservoirs. Therefore, understanding this hemoparasite transmission patterns can provide an insight into elevated disease risks, especially in the light of climate change, disappearance of wildlife species risks, and human disruption of natural ecosystems (Springer et al., 2015). Despite the involvement of different vectors, the sexual life cycle of both *Plasmodium* and *Babesia* species is completed in their respective vectors and shows significant overlapping stages (**Figure 1**). They start with gametogenesis in the midgut and end with sporozoites release in the salivary glands of the vector to allow the transmission to the hosts during blood meal with *Babesia * transmission mechanism resembling other hard tick-transmitted infections (Khan and Waters, 2004; Steere et al., 2016; Stewart and Rosa, 2018). A major difference in the transmission cycle of these protozoa is that all developmental stages of *Plasmodium* occur in adult *Anopheles* female mosquitoes, which are able to take multiple blood meals (Norris et al., 2010), while horizontal transfer from larvae to nymph or nymph to adult stages (transstadial transmission) is required for the completion of the *Babesia* sexual cycle in ticks. This is because each developmental stage of ticks takes blood meal only once, such that if a larva acquires the gametocytes of *Babesia* during initial blood meal, nymph will transmit sporozoites to the host during its blood meal to cause infection. Rarely, *Babesia* spp. also perpetuate in ticks by transovarial transmission (Stewart and Rosa, 2018; Jalovecka et al., 2019). Furthermore, the parasite transmission rates differ because mosquitoes use tube-like mouthparts called proboscis that can penetrate the host skin and suck up blood within seconds; however, ticks blood meal from the host is demonstrated to be slow and requires approximately 36 h during which the transmission of parasite occurs from the infected ticks.

*Plasmodium* begins its asexual cycle in the host by infection of liver hepatocytes by sporozoites delivered by the mosquito in the dermis [**Figure 1**, and (Gowda and Wu, 2018)], while *Babesia* appears to start its asexual cycle by direct invasion of erythrocytes by its sporozoites. Infection of the liver or any other organ by these protozoan sporozoites has not been documented. In the liver, *Plasmodium* undergoes a single round of replication producing ~10,000 merozoites in *P. vivax/P. ovale* and up to 30,000 in *P. falciparum* that are released by the lysis of hepatocytes (Antinori et al., 2012). Some *Plasmodium* spp. that infect primates, such as *P. vivax* and *P. ovale*, also form long-lived dormant parasites called hypnozoite (Markus, 2011) and often result in the relapse of malaria. This latent stage of *Plasmodium* can remain in the liver for more than a year (Vaughan and Kappe, 2017; Venugopal et al., 2020).

The obligatory intracellular life cycle in vertebrates enables both of these parasites to hide from the host immune system during most of their asexual reproductive stages. At this stage, the host immune response can only target the changed surface of the infected erythrocyte (Allred, 1995). In immunocompetent individuals, protective humoral and cellular immune responses together with innate immune response eliminate most of the parasites, but in the cases of immune deprivation, the infection can have dire consequences especially when not diagnosed and treated in a timely manner. In this review, using *Plasmodium* as a model system, because a significant body of literature is available for this parasite, we have compared the erythrocytic cycle of both pathogens in the hosts and summarized known differences and similarities in host immune responses during infection. We have also summarized in brief the mechanisms of immune evasion by *Plasmodium* species and the consequence of immunosuppression by both parasites on other pathogens.

## PATHOGENESIS

### Invasion of Erythrocytes

The name for apicomplexans comes from the characteristic apical complex of secretory organelles that are discharged in a tightly controlled and highly regulated order. In mature merozoites of both protozoa, different protein populations localize in the rhoptry bulb and neck, but the functions of many of these proteins still remain unknown. Rhoptries are twinned, club-shaped structures with a body or bulb region that tapers to a narrow neck as it meets the apical prominence of the merozoite. One group of such proteins called rhoptry-associated membrane antigen (RAMA) is indispensable for blood-stage parasite survival. RAMA is not required for the trafficking of all rhoptry bulb proteins but are essential for the invasion of red blood cells (RBCs) and do not induce cell membrane changes in target RBCs especially in the malaria parasite (Sherling et al., 2019). After emerging from the liver, *Plasmodium* merozoites invade RBCs. On the other hand, after tick bite, *Babesia* sporozoites appear to directly invade the erythrocytes, but proteins with function similar to RAMA are not shown in these parasites (Herwaldt et al., 2003).

Invasion of erythrocytes is an integral and essential component of both *Plasmodium* and *Babesia* life cycles. The intercellular adhesion molecule ICAM-4 is expressed on the surface of RBCs, and *P. falciparum* merozoites use it as one of their anchor points (Bhalla et al., 2015). At the same time, parasites secrete a subtilisin-like serine protease from their dense granules in order to modify the erythrocyte’s membrane and prepare it for invasion (Blackman et al., 1998). No evidence exists of *Babesia* using ICAM receptors; however, *B. divergens* uses neuraminidase- and trypsin-sensitive receptors such as glycophorins for cell invasion (Lobo, 2005). Bovine erythrocytes become rigid and adhere to vascular endothelial cells when infected with *B. bovis*. These modifications result in the appearance of ridge-like structures on the erythrocyte surface, similar to the knob-like structures observed on the surface of *P. falciparum* infected human RBCs, but they are morphologically and biochemically distinct (Hutchings et al., 2007). Interestingly, *P. vivax* and *P. ovale* prefer infecting reticulocytes (immature RBCs), which limits their replication and levels of parasitemia development (Lim et al., 2017; Kanjee et al., 2018; Pasini and Kocken, 2020), while *P. falciparum* infects both reticulocytes and mature erythrocytes (Pasvol et al., 1980; Srivastava et al., 2015) similar to that observed in canine pathogen *B. gibsoni*, which results in a high level of parasitic burden in blood and causes more severe disease. On the other hand, *P. malariae* prefers old erythrocytes, and human pathogen *B. microti* also exhibits a higher tropism for mature RBCs (Borggraefe et al., 2006). Nevertheless, these cellular and physiological changes are related to the virulence of both *Plasmodium* and *Babesia* (Hutchings et al., 2007).

### Intraerythrocytic Multiplication, Egress, and Re-Infection

**Figure 2:**
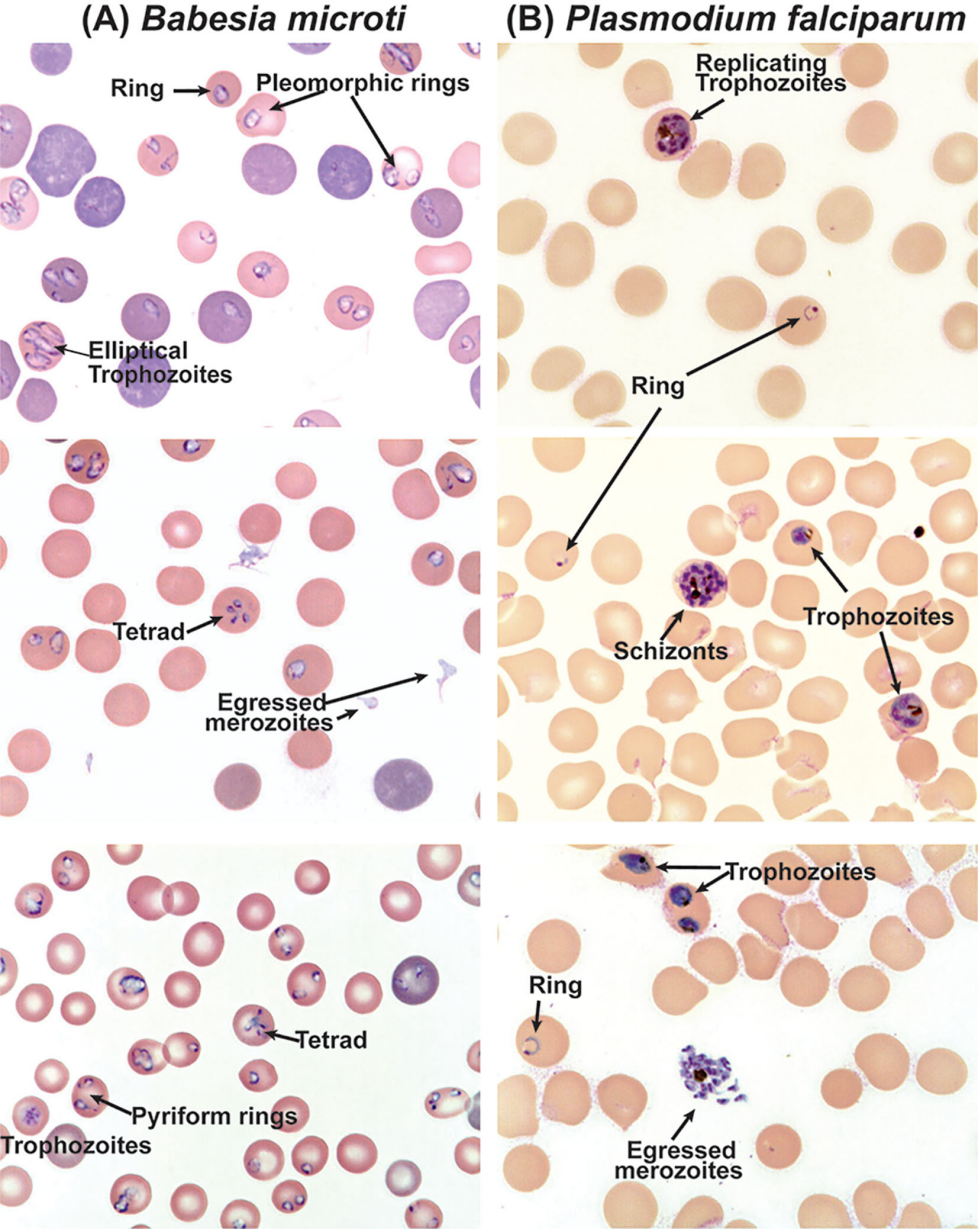
Differential morphological forms of *B. microti* during replication in RBCs of C3H mouse and *in vitro* culture of *P. falciparum*. **(A)** Clearly defined rings, pleomorphic trophozoites and pyriforms, and tetrads are observed during the replication of *B. microti*, while **(B)** rings, trophozoites, and schizonts are clearly observed during the erythrocytic cycle of *P. falciparum*.

Many host protein receptors have been shown to facilitate the adhesion and invasion of parasites; however, host molecules involved in interactions with the parasites during intracellular development remain poorly explored (Hentzschel et al., 2020). Even lesser is known about the *Babesia* interaction with RBCs. There are several differences in the erythrocytic cycle of *Plasmodium* and *Babesia*. Once the invasion of erythrocytes occurs, parasites rely on various host factors to grow and replicate. Repeated cycles of replication depicting different stages of infection: ring, trophozoites, and schizonts for *Plasmodium*, occur while schizogony has not been known for *Babesia* spp. (**Figures 1**, **2**). Despite the growing importance of this tick-borne disease, investigation of the basic biology of *Babesia* species that infect humans remains somewhat neglected. Its highly unusual variable intra-erythrocytic life cycle forms; the life span of each population of infected cells and the time required for the generation of the different stages of parasite have been documented to some extent. Unlike repeated replication cycles during schizogony to produce a large number of merozoites from a single RBC (~6-36/iRBC depending on *Plasmodium* species) (Antinori et al., 2012), only one to three *Babesia* replication cycles are reported to occur producing different morphological forms: paired figures, multiple trophozoites, pyriform, figure eight, Maltese Cross, etc. [**Figure 2** and (Cursino-Santos et al., 2016)]. Importantly, the choice of developmental pathway of these parasites is determined by the availability of RBCs for infection and nutritional environment present. Thus, parasites respond swiftly to the availability of uninfected RBCs for invasion and nutritional components needed (Cursino-Santos et al., 2016). 

Interestingly, malaria parasites replicate within the non-phagosomal parasitophorous vacuole (PV) and retain *Plasmodium* Translocon for Exported Proteins (PTEX) inserted in the parasitophorous vacuole membrane (PVM) to both release molecules into the erythrocytic cytoplasm and import nutrients (Ho et al., 2018). PTEX is used to export proteins in the RBC cytoplasm that remodels erythrocytes to facilitate the uptake of nutrients and the disposal of waste products (de Koning-Ward et al., 2016; Kalanon et al., 2016). In addition, PTEX is also involved in the modification of the erythrocyte membrane and the insertion of *Plasmodium* virulence proteins and adhesins, such as the highly variable *P. falciparum* erythrocyte membrane protein 1 (PfEMP1) in knob-like protrusion and ring-infected erythrocyte surface antigen (RESA) and different chaperons into the erythrocyte membrane. Although the parasite-derived multicopy variant erythrocyte surface antigen (VESA) proteins of *B. divergens, B. bigemina*, and *B. bovis* are also displayed on the surface of iRBCs, only three similar genes are present in *B. microti* and it is not known if they are even present on the infected erythrocytes surface (Cornillot et al., 2012; Jackson et al., 2014). Another major difference between *Plasmodium* and *Babesia* is their reaction to heme, a product of hemoglobin destruction that occurs during the invasion of RBCs. Heme is toxic for *Plasmodium* but has no effect on *Babesia* parasites. Heme is transported to the digestive vacuole of the *Plasmodium* where, by still an unclear mechanism, it is transformed into the hemozoin crystal, the characteristic pigment of this protozoan (Kapishnikov et al., 2021).

During rigorous asexual multiplication cycles, the rupture of PVM occurs and ultimately mature schizonts burst open the erythrocytic membrane to facilitate *Plasmodium* merozoites egress. The replicative cycle of *P. falciparum* followed by full development of invasive parasites is synchronized with their egress. Using inhibitors of known proteases, several parasite proteases have been identified as key effectors of the egress process for the blood stage as reviewed previously (Singh and Chitnis, 2017), in addition to perforin-like proteins with homology with mammalian perforins (PfPLP) and the subtilisin-like protease, PfSub1, that are located in micronemes and exonemes, respectively. These enzymes cause the disruption of the RBC cytoskeleton and the rupture of different membranes (Salmon et al., 2001; Millholland et al., 2011). Several of these effectors are localized in the apical organelles of apicomplexan protozoa and are secreted systematically to initiate the egress of merozoites. Furthermore, phosphoproteomic analysis of merozoites with a cGMP-dependent kinase inhibitor, compound 1, suggests the involvement of this enzyme in regulating the key processes of the invasion and egress of *P. falciparum* merozoites (Alam et al., 2015).

Although the *B. microti* egress mechanism has not been well documented until now, the release of *B. divergens* merozoites starts by establishing contacts with the plasma membrane of the erythrocyte from inside before they exit the cell (Sevilla et al., 2018). The merozoites released from iRBCs are ready for invasion of new erythrocytes and repeat multiplication cycle. In both protozoa, a small proportion of parasites commit to produce sexual progeny represented by gametocytes. Such commitment levels vary between species within the same genus and are affected by genetic, epigenetic, and environmental conditions. Once matured, these gametocytes are ready by the vectors to take up to start the sexual cycle in the midgut of the respective vectors.

## Immunity Against These Two Protozoan Pathogens

During the infection of hosts with parasites, a tug of war ensues to determine if a person gets seriously sick or recovers. Using the rodent malaria parasite *P. chabaudi* as a model system, Wale and coworkers presented three components of the immune response on distinct effects on parasites and disease (Wale et al., 2019). According to them, the host deploys a strategy in which it destroys RBCs early in infection to restrict erythrocyte supply for invasion. They attributed the loss of total RBCs to the indiscriminate removal of both iRBCs and uninfected RBCs causing anemia. This phenomenon of immunopathology appeared to be beneficial to the host by restricting the *Plasmodium* population. Late in the infection, the accelerated turnover of RBCs allowed mice to recover from anemia while simultaneously limiting *P. chabaudi* invasion and proliferation in erythrocytes. Ultimately, by returning of the components of the host response with respect to the resupply of reticulocytes and indiscriminate killing to the initial phase and infections taking different trajectories, heightened targeted killing response to iRBCs resulted in the decrease in parasite numbers to undetectable levels (Wale et al., 2019). A somewhat similar response was also observed when *B. microti* infected C3H mice (Djokic et al., 2018a; Djokic et al., 2019).

### Innate Immune Response

*Plasmodium* spp. challenge the mammalian immune system with a delicate balancing act. These parasites have acquired immune escape mechanisms that prevent the development of sterile immunity. During the past two decades, there has been a significant progress in understanding the molecular and cellular mechanisms of host–parasite interactions and the associated signaling of immune responses to the malaria parasite. Soon after infection, both the liver and blood stage parasites are sensed by various receptors involved in the host innate immune system resulting in the activation of signaling pathways and the production of cytokines and chemokines. Similar to other pathogens, innate immunity is the first line of defense against *Plasmodium* infection and starts during the initiation of the host–parasite contact. In the mouse model, *Plasmodium* sporozoites injection into the dermis recruits mast cells at the site of infection (Wilainam et al., 2015). Sporozoites make their entrance into liver hepatocytes by passing through blood circulation where liver resident Natural Killer (NK) and NKT cells are activated. During this stage, *Plasmodium* RNA likely stimulates pro-inflammatory type I interferon (IFN-I) in an interferon-α/β receptor-dependent manner and also stimulates the production of IFN-γ to facilitate phagocytosis by macrophages (Liehl et al., 2014).

Appropriately regulated innate immune response and pro-inflammatory cytokine production are required both for generating protective immunity and stimulation of adaptive immune response. Although in the early erythrocytic cycle, dendritic cells (DCs) and macrophages are key responders, macrophages have been known to become immunosuppressive after the phagocytosis of *Plasmodium* iRBCs while DCs continue to produce pro-inflammatory cytokines and chemokines to promote innate and adaptive immune responses (Schwarzer et al., 2003; Wu et al., 2015). In addition to producing a wide range of pro-inflammatory cytokines, including TNFα, IL-12, and IL-6, and chemokines, such as CXCL1, CXCL2, CCL2, CCL5, CXCL9, and CXCL10, during the *Plasmodium* erythrocytic cycle, DCs also contribute significantly for immunity development and pathogenesis of the parasite (Gowda and Wu, 2018; Wu et al., 2018). For example, DCs produce IL-12 that activates NK cells to induce the production of IFN-γ, which primes macrophages and neutrophils to enhance phagocytosis and promotes Th1 and effector T cell responses connecting innate to adaptive immune responses against the malaria parasite (Walsh and Mills, 2013; King and Lamb, 2015). During the *Plasmodium* infection, DC function is compromised to some extent that results in the reduction of immune responses against the parasite as well as against heterologous antigens.

*Babesia* susceptible C3H mice show several human-like disease manifestations and are ideal to study the pathogenesis of this protozoan. In the immunocompetent C3H mice, peak *B. microti* parasitemia of >40% was associated with a significant decrease in the hemoglobin level (Djokic et al., 2019). Splenomegaly and destruction of the marginal zone in spleen, and lysed erythrocytes and released *B. microti* life forms were also observed in these mice. After undetectable parasitemia was achieved, a significant increase in splenic macrophages levels was observed in *B. microti* infected mice suggesting a role of these phagocytes in disease resolution. The severe combined immunodeficient (SCID) mice as well as IFNγ-deficient mice with chronic *B. microti* infections demonstrated protective responses comparable to those of fully immunocompetent animals (Li et al., 2012). Furthermore, NK cell depletion *in vivo* did not significantly impair the protective responses. Conversely, macrophage depletion resulted in increased susceptibility to *B. rodhaini* infection that was associated with changes in their antibody and cytokine profiles, indicating a crucial role of macrophages in the protection against the challenge infection (Li et al., 2012).

Bastos et al. (2007) characterized peripheral blood monocyte-derived DC from *B. bovis* infected cattle, and myeloid DC from afferent lymph, but not resident DC from other bovine tissues (Bastos et al., 2007). For the hemoprotozoan infections like in babesiosis, the spleen as a secondary lymphoid organ is central to the innate and acquired immune response. The authors examined the phenotypic profile of myeloid DC from spleen and another myeloid cell population with monocyte features represented by the CD13+CD172a+/-CD14-CD11a-CD11b+/-CD11c+ and CD13+/-CD172a+CD14+CD11a-CD11b+/-CD11c+, respectively. (Bastos et al., 2007). The CD13+ population was obtained only from the spleen; however, they obtained the same percentage of CD172a+ population from the spleen and peripheral blood. The myeloid splenic DCs exhibited immature state. Early interactions of cells of the innate immune system including DCs and NK cells can influence the development and ability of the adaptive immune response to reduce/eliminate intracellular pathogens. Upregulation of MHCII, CD80, and CD86 in activated DCs during infection suggested that mature *Babesia*-activated CD13(+) splenic DCs were required for the stimulation of bovine NK cells (Bastos et al., 2008).

*Plasmodium* glycosylphosphatidylinositol (GPI) used to anchor several proteins into the merozoite membrane are structurally heterogeneous lipid molecules based upon differences in length and extent of unsaturated acyl residue presence and location of acyl components in the phosphatidylinositol moiety. GPI has been shown to induce the production of pro-inflammatory cytokines by macrophages primarily through recognition by TLR2-TLR1 heterodimer depending on GPI composition and by participation of TLR4 to somewhat lesser extent (Mockenhaupt et al., 2006; Gowda, 2007; Seixas et al., 2009; Zhu et al., 2011). This signaling likely occurs due to the burst of release of a large number of merozoites (up to 36/iRBC) of *Plasmodium* during the active erythrocytic cycle making GPI anchored proteins easy access to TLR2 and TLR4. Other TLRs, including TLR7 and TLR9, are also involved in signaling by RNA, DNA, and other molecules of the malaria parasite to produce pro-inflammatory cytokines and IFN-I (Gowda and Wu, 2018). Only limited information is available for GPI anchored merozoite proteins of *Babesia* species, and it was reported not to induce the stimulation of pro-inflammatory cytokines (Delbecq et al., 2002; Debierre-Grockiego et al., 2019; Nathaly Wieser et al., 2019). Unlike *Plasmodium* species, the composition of GPI in *B. microti* proteins has not been described yet, such that the contribution of TLR2 and TLR4 remains to be investigated to understand the influence of the signaling mechanisms during infection of the host defective in the specific TLRs. The role of TLR4 in *B. microti* pathogenesis is unknown, but its GPI anchors were suggested to possibly activate TLR4 and TLR2 (Campos et al., 2001; Debierre-Grockiego et al., 2007). Other TLRs, including TLR7 and TLR9, are also involved in signaling by RNA, DNA, and other molecules of the malaria parasite to produce pro-inflammatory cytokines and IFN-I (Gowda and Wu, 2018). Using TLR2 and TLR4 transfected human embryonic kidney (HEK) 293 cells, we observed the lack of production of pro-inflammatory IL-8 and TNFα cytokines when *B. microti* infected mouse blood or released parasites were used for stimulation (Akoolo et al., 2021). Different structure of GPI, the absence of *Babesia* GPI proteins display on the iRBCs surface, and a small number of released merozoites (2-8/iRBC) could all account for the absence of TLR2/TLR4 stimulation by *B. microti* merozoites/iRBCs. Lyme disease causing *Borrelia burgdorferi*, which is also transmitted by ticks, expresses large amounts of TLR2 ligand lipoproteins. This treatment served as a control to show the stimulation of TLR2 and to less extent TLR4 to increase pro-inflammatory cytokine IL-8 and TNFα production significantly (Akoolo et al., 2021). The involvement of host TLR2 during Babesia infection is not yet examined. 

#### Adaptive: Cellular Immune Response

Adaptive immune response is also dependent on the *Plasmodium* stage of the life cycle. Although antibodies appear to be important during erythrocytic infection, T cells are the major immune modulators in the liver and erythrocytic stages of infection. CD8+ cells specific for *Plasmodium* antigens eliminate the intracellular parasite in hepatocytes by NO and IFNγ-dependent and independent mechanisms (Doolan and Martinez-Alier, 2006; Chakravarty et al., 2008; Kelemen et al., 2019). Involvement of CD8^+^ T cells was also shown during the *P. vivax* erythrocytic cycle (Burel et al., 2016; Junqueira et al., 2018). During both cellular and humoral immune responses, CD4+ cells carry out their traditional helper function (Perez-Mazliah and Langhorne, 2014; Soon and Haque, 2018). Inflammatory CD4+ T-cells that produce IFNγ and TNFα have been implicated in conferring protection from malaria in adults by preventing *P. falciparum* infection or its replication such that higher parasitemia and clinical malaria are diminished (Boyle et al., 2015). In a study involving children in Uganda that is endemic for *P. falciparum* infection, Boyle and coworkers showed that although all children exhibited similar percentage of CD4+ cells as adults and uninfected children, somewhat lower levels of *P. falciparum* specific CD4+ T cells producing IFNγ, IL-10, and TNFα cytokines were present in infected children’s blood with asymptomatic malaria (Boyle et al., 2017). IL-10 secretion by CD4+ cells diminished with the age of the individuals. They also showed a high positive correlation between parasite burden and the frequencies of *P. falciparum-*specific IL-10 producing CD4+ T cells but not with IFNγ or TNFα producing CD4+ cells; however, these cells could not prevent infection prospectively. Importantly, IL-10 exerted quantitatively stronger regulatory effects on innate and CD4+ T cell responses during primary and secondary infections, respectively. The durability of IL-10-producing CD4+ T cells post-infection demonstrated how this cytokine may contribute to optimizing the control of parasites and prevent immunity-mediated pathology during recurrent malaria infections (Villegas-Mendez et al., 2016).

Cell-mediated immunity appears to be critical in preventing severe blood-stage malaria. IFNγ, but not IL-2, was shown to play an essential role in the cell-mediated immunity against *P. chabaudi adami* infections (Batchelder et al., 2003). The inability to generate protective immune response against *Plasmodium* spp. and inadequate T-cell responses could help persistent blood-stage infection and malaria. The lack of effective protection against malaria albeit production of highly inflammatory cytokines during infection suggests the absence of adequate memory T cells development to confer immunity. The *Plasmodium* ortholog of the macrophage migration inhibitory factor was found to enhance inflammatory cytokines production and also induced antigen-exposed CD4+ T cells to mature into short-lived effector cells and not into precursor cells for memory development. These findings indicate that the parasite actively interferes with immunological memory development and could have helped maintain the conserved orthologs of the parasite macrophage migration inhibitory factor during evolution (Sun et al., 2012). The lack of immunological memory could account for failure of various vaccine candidates to confer protection against malaria.

*B. microti* strain KR-1 was isolated from a Connecticut resident with babesiosis. The inoculation of this strain in SCID mice and TCRαβ knockouts resulted in the sustenance of severe but nonlethal parasitemia averaging 35% to 45% infected erythrocytes (Clawson et al., 2002). IFNγ-deficient mice developed a less severe parasitemia and were able to clear infection. In contrast, in six of eight JHD-null mice (B-cell deficient), the levels of parasitemia were indistinguishable from those in the wild-type animals. To summarize, these results indicate that cellular immunity is critical for the clearance of *B. microti* in BALB/c mice and that disease resolution can occur even in the absence of IFNγ (Clawson et al., 2002).

Depletion of CD25+ Treg cells significantly reversed the inhibition of CD4+ T-cell proliferation and IL-2 production, indicating that this cell population contributes to the suppression of T-cell function during malaria (Nie et al., 2007; Wammes et al., 2013). Moreover, depletion of Treg cells prevented the development of parasite-specific Th1 cells involved in the induction of cerebral malaria during a secondary parasitic challenge, demonstrating a regulatory role for this cell population in the control of pathogenic responses that otherwise lead to fatal disease. As mentioned above, immune responses against *Plasmodium* protozoan are unable to inhibit the establishment of its infection and can even contribute to the severity of disease. Individuals who survive multiple infections show some level of protective immunity where primed immune responses inhibit severe disease manifestations by limiting inflammation. CD4+ T cells play critical roles in the immunoregulatory pathways established during malaria by priming for phagocytosis mediated killing of parasitized RBCs, and by helping B cell differentiation to produce functional anti-*Plasmodium* antibodies (Kumar et al., 2020). In malaria-endemic areas, a segment of population that shows minimal clinical signs, if any, could become chronic carriers of Plasmodia. The Tregs activation marker TNFRII expression was shown to increase during the infection of these individuals but diminished after treatment. This TNFRII expression showed positive correlation with TNFα in response to *P. falciparum*-infected RBCs, but this association disappeared after treatment. Asymptomatic malaria also appeared to be associated with increased TNFRII expression on Tregs, together with Th2 cytokine responses suppression, which could facilitate the survival of the parasites in *Plasmodium* carriers (Wammes et al., 2013). The apparent suppression of IL-13 secretion in response to *P. falciparum*-infected erythrocytes also recovered after the treatment of individuals for malaria.

Immunocompetent mice mount a very strong adaptive immune response against *B. microti*, with features including the following: (i) a robust germinal center induction; (ii) follicular helper T cell development that accounts for ~30% of splenic CD4+ T cells; and (iii) an increase in effector T-cell cytokine levels, including IL-21 and IFNγ together with enhanced secretion of specific antibodies (Yi et al., 2018). Interestingly, parasitemia of *B. microti* was significantly lower in the Towns sickle cell disease mouse strain. Although splenic architecture is highly disorganized before infection in these mice, they elicited a surprisingly robust adaptive immune response, including comparable levels of germinal center B cells, follicular helper T cells, and effector cytokines, and higher immunoglobulin G responses against potential immunogenic epitopes of two *Babesia*-specific proteins.

*B. microti* infected C3H mice also showed significantly higher concentration of CD4+ cells secreted cytokines such as IL-2 and IFNγ in plasma, while the increase of cytokines such as IL-4, IL-5 and IL-13 secreted by CD8+ cells was not always significant. (Djokic et al., 2019). Thus, Th1 cells-mediated immunity appears to be important in the clearance of this intracellular pathogen. A significant increase in IL-6, which induces Th17 cell differentiation, was observed; however, it led to only a moderate change in Th17 cell secreted cytokines, IL-17A, IL-17F, IL-21, and IL-22. A similar immune response to *Trypanosoma* infection was reported to affect the clearance of this parasite, as well as of coinfecting pathogen(s) (Hoft et al., 2000; Santamaria and Corral, 2013; Cai et al., 2016). At the acute stage of *B. microti* infection, splenic cells exhibited Th1 polarization in young mice with an increase in IFNγ and TNFα producing T cells and a simultaneous increase in the Tregs/Th17 ratio. These changes likely help in the clearance of infection in young mice and also prevent mortality that occurs due to the infection of mice with *B. duncani* WA-1 strain that stimulates potent inflammatory response infection in mice (Djokic et al., 2018b).

The role of T cell subpopulations in protective cellular immunity development at the early stage of infection with *B. microti* and *B. rodhaini* was depicted by the changes in the course of infection and delayed type hypersensitivity (DTH) response against parasites (Shimada et al., 1996). Lyt-2+ and L3T4+ T cells showed opposite effects on *B. microti* and *B. rodhaini*. Depletion of Lyt-2+ T cells increased resistance to *B. microti* and susceptibility to *B. rodhaini* infection. In contrast, depletion of L3T4+ T cells enhanced susceptibility to *B. microti* infection but led to increased resistance to *B. rodhaini* infection. The DTH response to *B. microti* in infected mice was exacerbated by depletion of Lyt-2+ T cells but reduced significantly after L3T4+ T cell depletion. A self-limiting infection of *B. microti* in mice still makes them resistant to reinfection. Anti-CD4 monoclonal antibody (MAb) treatment of immune mice restricted the protective immune response against challenge infection; however, anti-CD8 MAb treatment did not affect protection. Supporting these results, the transfer of CD4+ T-cell-depleted spleen cells resulted in higher parasitemia than when CD8+ T-cell-depleted spleen cells were injected. An increase in IFNγ production by CD4+ T cells was observed in the culture supernatant of spleen cells from immune mice. Treatment of immune mice with anti-IFNγ MAb reduced their protection from *B. microti* infection to some extent. Furthermore, protection against challenge infection was not observed in IFNγ-deficient mice. On the other hand, treatment of immune mice with MAbs against IL-2, IL-4, or TNFα did not affect protective immunity. Although parasitemia of *B. microti* declined in CD4-deficient mice, these mice maintained parasites for more than a month (Skariah et al., 2017). Treatment of mice with anti-CD4 MAbs and transfer of naïve mice with CD4^+^-depleted spleen cells showed significantly higher parasitemia after challenge infection (Igarashi et al., 1999). These combined results suggest the essential requirements for CD4+ T cells and IFNγ in protective immunity against challenge infection with *B. microti*.

Infection with *B. duncani* strain WA1 is fatal in mice, whereas *B. microti* parasitemia gets resolved and the latter infection is not fatal. Mortality due to WA1 inoculation is associated with the stimulation of high levels of TNFα production, whereas the resolution of *B. microti* infections was associated with an increase in IL-10 and IL-4 production. The contribution of excessive TNFα production was further emphasized by using TNFRp55-/- mice that exhibited a 90% survival rate due to the disruption of the TNFα−stimulation pathway resulting in diminished pathology associated with WA1 infection. Thus, a high level of TNFα produced by mice is an important mediator of the WA1 pathogenesis (Hemmer et al., 2000). WA1 infection in T-γδ^-/-^ cells and wild-type mice also resulted in fatality. CD4+ T cells participate in parasite elimination during *Babesia* infection, while CD8+ T cells may also contribute to the disease manifestations by the WA1 strain.

DCs are the bridge between innate and adaptive immunity such that interactions between antigen-presenting DCs and inducible T cells are necessary for the stimulation of an adaptive immune response. During *Plasmodium* infection, DC function is attenuated due to hemozoin released from engulfed iRBCs resulting in the reduction in immune responses against both parasitic and heterologous antigens. These results suggest that the suppression of both DC costimulatory activity and functional T cell responses reduces the immunity against *Plasmodium* (Millington et al., 2007; Hisaeda et al., 2008; Yap et al., 2019). Supporting this premise, TLR9(-/-) mice infected with *P. yoelii* were somewhat resistant to fatal infection through limited activation of Tregs that impaired effector T cell development (Hisaeda et al., 2008). Additionally, DCs of humans inflicted with malaria mediated strong immunosuppression through the induction of Indoleamine 2,3-Dioxygenase 1(IDO1) and Lymphocyte Activation Gene 3 (LAG3), which attenuates inflammatory response by decreasing class II antigen presentation (Vallejo et al., 2018). Furthermore, *P. vivax* downregulates three G-protein coupled receptors, CXCR1, CXCR2, and CSF3R, that induces the depletion of neutrophil populations. While the immunosuppressive signaling has been reported during infection with different *Plasmodium* spp., such a response to *P. falciparum* was significantly more pronounced (Vallejo et al., 2018). CD40 responses to merozoites and pro-inflammatory cytokine production by DCs were impaired in the presence of freshly isolated *P. falciparum* infected RBCs (Yap et al., 2019). Similarly, after *P. berghei* ANKA infection clearance, robust immunological memory against malaria parasites was reported, albeit the splenic DCs had significantly decreased the capacity of cytokine production as well as lower surface expression of MHC Class II molecules (Adachi and Tamura, 2020).

Compared to replication-deficient parasites, immunization with replication-competent parasites confers better protection and also induces an IFN-I response, but whether this response has beneficial or adverse effects on vaccine-induced adaptive immunity is not known. When mice deficient in IFN-I signaling were immunized with replication-competent sporozoites of rodent parasite *P. yoelii*, they demonstrated superior protection against infection (Minkah et al., 2019). Strong CD8+ T cell memory response development (also in liver) showed correlation with this protection. In a complementary experiment, the adoptive transfer of memory CD8+ T cells recovered from the livers of IFN-I signaling-deficient immunized mice offered increased protection specifically against liver stage parasites. Overall, these results demonstrate that hepatic CD8+ T cell memory development is impaired by IFN-I signaling stimulated by the liver stage parasites. On the other hand, innate, cellular, and humoral immune responses conferred by IFNγ and Th1 type responses were documented to play critical roles in controlling blood-stage malarial disease. Inflammatory responses help the resolution of infection. In contrast, anti-inflammatory immunomodulators, TGFβ, and IL-10 were considered important in reducing inflammation and pathology during malaria (Drewry and Harty, 2020). The specific mechanisms and pathway involved in which TGFβ helps in offering protection against severity of malaria remain to be investigated.

During the murine modeling of severe malaria, conventional DCs (cDCs) lose the ability to phagocytose and present parasitic antigens when the burden of pathogen increases resulting in the suppression of CD4+ T cell responses. IFN-I signaling adversely affects cDC function, such that they are unable to fully prime Th1 cells that produce IFNγ cytokine. IFN-I signaling was shown to modulate all subsets of splenic cDCs; however, CD8-lacking cDCs were found to be most susceptible such that type I IFNs reduced their phagocytic and Th1 responses. In response to *P. berghei* infection, IFN-I signaling of cDCs resulted in rapid and substantial IFNα production, which also suppressed the Th1 response. Overall, results from Haque and coworkers suggest that the elimination of IFN-I signaling in CD8-negative splenic cDCs results in increased Th1 responses against *Plasmodium* and also against other pathogens that induce IFN-I signal (Haque et al., 2014).

#### Adaptive: Humoral Immune Response

Pre-erythrocytic stage antigens are good targets for the elimination of *Plasmodium* species by vaccines because the parasitic population is small, and the chance of generation of escape mutants is low. Several targets for antibodies include sporozoites circumsporozoite protein (CSP), LSA1, TRAP, and Apical Membrane Antigen-1 (AMA-1) (Marsh and Kinyanjui, 2006). The AMA-1 molecule is involved in the invasion of RBCs and has also been described as an excellent vaccine candidate. Interestingly, antibody production against the extracellular domain of *B. divergens* AMA-1 is weak and produced late, usually between 1 and 5 months of parasites post-inoculation even though both IgG1 and IgG2 were induced (Moreau et al., 2015). Human IgG antibodies to *P. falciparum* antigens PfEMP1 and RIFIN are sufficient to activate antibody-dependent cellular cytotoxicity by primary human NK cells, which highly selectively lyse iRBCs and inhibit parasite growth (Arora et al., 2018). However, mouse IgG against *P. yoelii* and *P. chabaudi chabaudi* bound poorly to the surface of parasitized RBCs and did not affect their clearance, despite the continuation of inflammation, albeit antibodies prevented the infection of the next RBC generation. Compared to other parasitic infections, while a direct role for IgA in malaria infection has not been investigated, IgG, IgE, and IgM were shown to contribute to the adverse pathology in the rodent model. Malaria parasites induce the development of specific IgA secreting B cells among individuals who had multiple episodes of infection (Deore et al., 2019). Optimal function of *Plasmodium*-specific IgG antibody, which strongly bound to surface molecules of merozoites released from the erythrocyte, needed contributions of splenic macrophages and dendritic cells for parasitic elimination (Akter et al., 2019). CD4+ T cell subtypes were reported to be associated with immune functions and production of different antibody isotypes (Oakley et al., 2014). T-bet can suppress *Plasmodium*-induced apoptosis or stimulate the proliferation of T cells and thus may be responsible for T-bet-dependent antibody isotype switching and lower parasite burden. Interestingly, during *P. vivax* infection, children develop higher levels of IgM but not IgG antibodies, while IgG3 are more prevalent in adults (Oyong et al., 2019).

Antibody participation is not essential for offering cross-protection against other hemoparasites. Infection of mice with *B. microti* could protect mice against a follow-up fatal infection by *B. rodhaini* (Efstratiou et al., 2020) by reducing parasitemia levels. Surprisingly, significant reduction in the level of antibodies was observed in the protected mice, and levels of cytokines including IFNγ, IL-2, IL-8, IL-10, and IL-12, and of nitric oxide after infection with *B. rodhaini* also diminished. Mice immunized with dead *B. microti* were not protected from *B. rodhaini* infection, although high antibody responses were induced indicating a minor role played by antibodies for protection against death. Inoculation with live *B. microti* also protected against subsequent fatal malarial infections in mice and primates. Unexpectedly, immunization with dead *B. microti* led to lethal *P. chabaudi* infection despite the induction of high antibody responses, which means that other products of parasite metabolism, not just the antigens, play an important role in the development of generalized immune response against these two apicomplexans. Notably, cross-protection was also observed in mice lacking functional B and T lymphocytes. On examination of the other innate immune effector cells, mice depleted of NK cells showing chronic *B. microti* infection were also protected from *P. chabaudi* infection (Efstratiou et al., 2020). In contrast, *in vivo* depletion of macrophages in mice made them susceptible to *P. chabaudi*. These results show that the cross-protection offered by one protozoan, *B. microti*, against another, *P. chabaudi*, is dependent on innate immune response and appears to rely mainly upon the macrophage function.

The molecules of *Plasmodium* driving humoral immunity include CD4+ T cells, B cells, and IL-21 together with the T cell costimulator. In *P. chabaudi chabaudi* AS infection, early IL-6 stimulation resulted in *Plasmodium*-specific IgM but not IgG production. IL-6, rather than germinal center B-cell differentiation, induced the splenic CD138+ plasmablast development. IL-6 also induced the T cell costimulator in CD4+ T cells causing their localization adjacent to splenic B cells. In addition, IL-6 promoted the control of parasitemia and induced IgM and IgG production, T cell costimulator expression by Tfh cells, and germinal center B-cell development. This cytokine also promoted the stimulation of CD4+ T cell and B cell responses during the erythrocytic cycle of *Plasmodium* that facilitated the production of protozoan-specific antibodies (Sebina et al., 2017).

To evade the host immune responses, *P. vivax* disrupts the function of the B cell subsets. Some antibodies produced during infection have the potential to block *Plasmodium* invasion as well as its dissemination into the host. The prevalence of antibodies to *P. vivax* merozoite surface protein-8 (PvMSP8) was reported to be high during and after infection. The anti-PvMSP8 antibody responses could be detected for 4 years in some patients who had recovered from an infection suggesting that persistence of long-term humoral immune response occurs against PvMSP8 (Kochayoo et al., 2019); however, no correlation of this response was observed with the presence of titer of circulating antibodies.

IL-10 is a pleiotropic cytokine expressed during malaria and is essential for anti-*Plasmodium* humoral immunity. Germinal center B cell reactions, isotype-switched antibody responses, parasite control, and host survival require B cell-intrinsic IL-10 signaling. IL-10 also indirectly supported humoral immunity by suppressing excessive IFNγ, which induces T-bet expression in B cells. B cell-produced IL-10 increased, whereas IFNγ and T-bet from B cells suppressed germinal center B cell responses and anti-*Plasmodium* humoral immunity (Guthmiller et al., 2017). *Plasmodium* infection-induced IFN-I limited T follicular helper accumulation and constrained malaria-specific humoral immunity. CD4 T cell associated IFN-I signal stimulated T-bet and Blimp-1 expression and induced T regulatory 1 responses. Secreted IL-10 and IFNγ cytokines of T regulatory 1 cells together restricted the accumulation of T follicular helper cells, restricted specific antibody production, and allowed the persistence of parasites. The authors suggested that IFN-I-mediated Blimp-1 induction that caused the expansion of T regulatory 1 cells is a systemic, inflammatory response to viral and parasitic infections and accompany humoral immune response suppression (Zander et al., 2016).

The effects of *P. falciparum* infection on peripheral B-cell subsets have been investigated, but limited information is available for *P. vivax*. In a prospective study with malaria patients to determine peripheral B cell profiles, the authors reported a temporary increase in atypical memory B cells and B cell-activating factor (BAFF)-independent percentage of total and activated immature B cells in malaria patients. An increase in TLR4 expression occurred in naive B cells from malaria patients. During acute infection, total IgM levels remained steady; however, a significant increase in these antibodies was observed at the recovery stage. Persistence of serum IgM antibodies specific to parasite proteins was observed during the recovery of patients (Patgaonkar et al., 2018).

*B. microti* infection resulted in high levels of IL-10 production due to increased frequency of IL-10-producing regulatory B cell and T cell presence. In contrast, the absence of B cells in B cell-deficient mice demonstrated increased susceptibility of these mice to *B. microti* infection (Jeong et al., 2012). The ability of immunized rats to resist challenge with *B. divergens* was suggested to be splenic independent (Ben Musa and Dawoud, 2004). The uptake of infected erythrocytes by the liver was suggested not to happen in immune rats. Rather the clearing of parasitemia in rats could depend upon antibody inhibition of merozoite invasion. Histological studies on livers collected from immune rats showed that lymphocytes are accumulated in the liver, and these consisted of B and T cells. Liver leukocytes might therefore be very important in the development of acquired immunity to *B. divergens* in splenectomized rats.

### Immune Evasion and Immunosuppression

Human complement is the first line of defense against invading pathogens, including the *Plasmodium* and *Babesia* parasites. The complement represents a particular threat for the clinically relevant blood stages of the parasites such that this protozoan has evolved to overcome its effects. To evade complement-mediated destruction, the parasites bind host factor H (FH) to FH-related protein 1 (FHR-1) or the specific receptors on their surface, thus competing host cells for binding, and thus resulting in the prevention of complement-mediated opsonophagocytosis. These proteins accumulate differentially on the surface of intraerythrocytic schizonts versus free merozoites. The purpose of two proteins (FHR-1 and specific receptor) displaying the same activity is poorly understood as the mechanisms involving both proteins target the same FH-mediated human immune response and facilitate similar evasion of complement-mediated killing (Reiss et al., 2018).

Erythrocytic rosetting is a mechanism involved in avoiding phagocytosis-mediated elimination of iRBCs. The surface of *Plasmodium*-infected RBCs is decorated with parasitic proteins, which are targeted by monocytes. Some of the parasitic proteins displayed on the iRBCs make them stickier. Type I rosetting is accomplished by direct interaction of ligands on iRBC with uninfected RBC receptors. Thus, the infected erythrocytes get surrounded by a protective cage of uninfected RBCs, which results in a formation called “rosettes.” Insulin like growth factor-binding protein 7 (IGFBP7), a protein secreted by monocytes in response to parasitic infection, stimulates rosette formation by *P. falciparum* and *P. vivax* infected erythrocytes. IGFBP7-mediated type II rosette formation was observed to be fast albeit a reversible process (Lee et al., 2020) and required von Willebrand factor and thrombospondin-1 serum factors. The interaction of these factors with IGFBP7 promotes rosette formation by the iRBC. Engulfment of iRBC by host phagocytes was reported to be hampered by the IGFBP7-induced type II rosette formation.

Although infection by *B. microti* is usually asymptomatic in immunocompetent young individual, persons aged >40 years often experience serious manifestations, and elderly exhibit life-threatening disease (Krause et al., 2003; Akoolo et al., 2017) because persistent parasitemia increases with the age of individuals and often their immune status is somewhat compromised. Since susceptible mice exhibit many symptoms similar to those observed in humans, they serve as a model system to investigate pathogenesis, immune response, and babesiosis disease. BALB/c and C57BL/6 mice were found to be resistant, regardless of age, which indicates that the genotype of mice determines the resistance to *B. microti* (Vannier et al., 2004). Unlike immunocompetent mice, SCID mice, which retain an innate immune system but lack the lymphocytes needed for adaptive immunity, were shown to develop high and persistent parasitemia. Of importance, mice also showed age-associated loss of protection such that when spleen cells were transferred from older immunocompetent mouse strain (18 months old), cells from BALB/c mice, but not DBA mice, were able to control persistent parasitemia in SCID mice. Thus, the age (as observed in humans) and genetic make-up of the donor of splenic cells determined the adequate building up of the protective immune response in young recipient SCID mice, and hence, cells from older mice adversely affected the control of persistent parasitemia in immunocompromised mice (Vannier et al., 2004).

During their asexual stages in vertebrate hosts, *Plasmodium* parasites mainly hide in the hematopoietic colonies of the bone marrow (Venugopal et al., 2020). For infection, *P. malariae* shows preference for mature erythrocytes and *P. vivax* to immature RBCs. *Babesia* species also prefer the invasion of the mature RBCs (Borggraefe et al., 2006). Sequestration of *P. falciparum*-infected RBCs by cytoadherence helps in immune evasion and prevents their clearance by spleen. Different variants of the *P. falciparum* protein PfEMP1 that are displayed on iRBCs facilitate their binding to various receptors including CD36 on endothelial cells causing the sequestration of parasites in different organs. Furthermore, host endothelial receptors ICAM1 and endothelial protein C receptor (EPCR) are used for sequestration in the brain and cause cerebral malaria (Smith et al., 2000), whereas chondroitin sulfate A binding by some variants (Var2CsA) facilitates placenta binding (Fried and Duffy, 1996). This sequestration allows parasites to hide in the brain, lungs, liver, intestine, dermal tissues, and placenta, thereby avoiding splenic clearance of iRBCs. Adherence-mediated sequestration could also cause persistence of infection with potential for fatal consequences due to cerebral malaria and as a result of single and multiple organ failure of kidneys, liver and lungs. Cerebral malaria has been reported to occur due to iRBC sequestration within the brain blood vessels and is exacerbated by inflammation caused by the stimulation of the effector cells including T lymphocytes and the production of pro-inflammatory cytokines in the host. Sequestration of *Plasmodium* spp. by adherence to chondroitin sulfate 4 present on placental cells could result in congenital malaria (Fried and Duffy, 1996; Nunes and Scherf, 2007; Fried and Duffy, 2017; Fried et al., 2017).

The suppression of the host immune system by parasites plays critical roles on the persistence of infection. During acute malaria infection with both *P. falciparum* and *P. vivax*, significant reduction in lymphocyte counts was observed in the peripheral blood in Ethiopia [**Table 1**; (Kassa et al., 2006)]. This diminished immune status could make a patient susceptible to other infections that may occur simultaneously with malaria resulting in serious consequences. In fact, coinfection of mice with *P. berghei* and relapsing fever spirochete *B. duttonii* led to decreased malaria parasite burden but enhanced levels of relapsing fever spirochetes and an increased death rate (Lundqvist et al., 2010). Since C3H mice are susceptible to *B. microti* and show symptoms like parasitemia, anemia, splenomegaly, and hepatomegaly, suppressive impact on splenic lymphocytes (**Table 1**) could be extrapolated to also occur during infection of humans (Krause et al., 1996). In fact, coinfection of *B. microti* with Lyme spirochete, *Borrelia burgdorferi*, in C3H mice resulted in a significant increase in the burden of spirochetes in different organs and resulted in the persistence of inflammatory Lyme disease manifestations (Djokic et al., 2019) similar to that observed during *Plasmodium–Borrelia* coinfections. **Table 1** shows that *B. microti* subverted the splenic immune response, and a marked decrease in splenic B and T cells also resulted in the reduction of the levels of antibodies and hence diminished functional humoral immunity. Furthermore, increased *B. burgdorferi* burden in organs and severe inflammatory Lyme disease manifestations resulted due to immunosuppression by *B. microti* in coinfected mice showed an association with enhanced Lyme disease manifestations (Djokic et al., 2019). A similar response was also reported with respect to *Mycobacterium tuberculosis* coinfection with rodent malaria parasite *P. yoeli* (Blank et al., 2016).

**Table 1:** Suppression of adaptive immune response during *Plasmodium* and *Babesia* infection.

Cell Type	Absolute count/μl of blood (Mean ± SD) in humans^a^		
	Control (N=46)	*P. vivax* (N=69)	*P. falciparum* (N=7)
Lymphocytes	1,815 ± 729	1,078 ± 583	940 ± 472
T cells (CD3+)	1,379 ± 607	819 ± 404	701 ± 378
CD4+ cells	691 ± 234	455 ± 240	387 ± 206
CD8+ cells	643 ± 482	336 ± 200	297 ± 203
B cells (CD19+)	192 ± 98	86 ± 56 (N=43)	61 ± 39 (N=26)
	**Mean splenocytes count in young C3H mice infected with *B. microti*^b^**		
	**Control**	***B. microti* (acute phase)**	***B. microti* (after parasitemia resolution)**
T cells (CD3+)	9,220/8,710	14,250 ± 670	8,590 ± 1,160
CD4+ cells	4,900/4,900	4,920 ± 300	1,880 ± 180
CD8+ cells	3,900/3,200	1,139 ± 140	480 ± 280
B cells (CD19+)	18,150/19,610	27,910 ± 1,070	12,590 ± 2,680

P. falciparum shows severe disease compared to P. vivax and has more pronounced effect on adaptive immune response of humans during infection.

^a^(Kassa et al., 2006).

^b^(Djokic et al., 2019).

## Disease and Symptoms

In addition to HIV/AIDS and tuberculosis, malaria is considered as one of the three major fatal diseases in the world (Medaglini and Hoeveler, 2003). Therefore, WHO Global Malaria Programme, with active participation of NIH and Gates Foundation, provides technical support to malaria-endemic countries to attain the goals of reducing malaria cases and mortality by 90% in 2030. Malaria is primarily a tropical disease, and symptoms associated with the erythrocytic cycle of *Plasmodium* spp. usually occur during rainy season when mosquitoes are active. A symptomatic malaria patient could exhibit cough, rapid heart rate and breathing, fatigue, and overall malaise in addition to usual fever, chills (despite high surrounding temperature), headache, etc. Persistence of infection during asymptomatic malaria was reported to occur in Mali where only transmission season shows association with the symptomatic malaria (Andrade et al., 2020). During the dry season, PCR positive individuals showed subclinical *P. falciparum* infection that was more pronounced in older children and young adults than young children. Protozoa isolated during the dry season were transcriptionally and metabolically distinguishable from those recovered from persons with febrile malaria during the rainy, active transmission season, and the host immune cells and inflammation markers in these asymptomatic individuals were similar to those in uninfected controls. The parasites during the dry season also showed poor cytoadherence allowing increased splenic clearance (Andrade et al., 2020).

Cerebral malaria is the most severe complication of human infection with *P. falciparum*, but the mechanisms involved are still not fully understood. Pro-inflammatory immune responses are required for the control of blood-stage malaria infection but are also implicated in the pathogenesis of severe cerebral malaria. A fine balance between pro- and anti-inflammatory immune responses is required for parasite clearance without the induction of host pathology. The most accepted experimental model to study human cerebral malaria is *P. berghei* ANKA (PbANKA) strain infection in C57BL/6 mice that leads to the development of a complex neurological syndrome, which shares many characteristics with the human disease. In a study by Blank and coworkers, *M. tuberculosis* coinfection did not change the clinical trajectory of PbANKA-induced experimental cerebral malaria (Blank et al., 2016). In fact, the immunological environments in spleen and brain were similar in singly infected and coinfected mice. Overall levels of cytokine production and T cell responses in coinfected mice were also similar to PbANKA infected animals when inoculated alone. Another rodent parasite, *P. yoelii* coinfection with *M. tuberculosis*, resulted in a slight increase in *M. tuberculosis* burden detected by measuring lung CFU in coinfected mice and exacerbation of tuberculosis manifestation that also coincided with elevated levels of both pro-inflammatory (levels of IFNγ, IL-6, TNFα) and anti-inflammatory (IL-10) responses. Enhanced T cell responses in coinfected mice likely contributed to increased cytokine production (Blank et al., 2016). Malaria parasite immunosuppressive effects may also play a role on enhancing the severity of *M. tuberculosis*-induced pathology in mice, similar to that observed during *B. microti–B. burgdorferi* and *P. berghei–B. duttonii* coinfections.

Pregnant women and children below 5 years of age remain most susceptible to malaria because *Plasmodium* is capable of vertical transplacental transmission from mother to child. As a result, another devastating consequence of *P. falciparum* infection in pregnant women is congenital malaria that remains a major global problem especially in the endemic regions (Bilal et al., 2020; Danwang et al., 2020). Congenital malaria is defined as the presence of intraerythrocytic malaria parasites in the cord blood and/or the peripheral blood of an infant within the first week of birth, independent of display of the clinical symptoms (Omer et al., 2020). Infected newborns often show nonspecific, sepsis-like clinical manifestations in which early treatment can result in diminished risk of complicated malaria. Based upon the meta-analysis of 1,961 studies, the congenital malaria level was reported to be ~40%, and difference between prevalence of cases in continent of Africa and outside Africa was not statistically significant (Danwang et al., 2020). In some newborns, clinical malaria was observed within 7 days of birth (40/1,000); however, a few showed disease until 28 days of birth (10/1,000). Relatively lower prevalence of malaria in newborns was attributed to the presence of high concentration of fetal hemoglobin in neonatal red blood cell that provided unfavorable conditions for *Plasmodium* replication and growth, with transfer of maternal immunity also playing a role in protection. Placental *Plasmodium* infections were found to show significant association with the risk of perinatal morbidity and mortality such as preterm birth, low birth weight, and intrauterine fetal loss (Osungbade and Oladunjoye, 2012). The prevalence rate of congenital malaria is usually significantly higher in non-endemic regions (Quinn et al., 1982; Bilal et al., 2020). Using *P. falciparum* infected placental samples, Duffy et al. described that iRBCs obtained from placenta bound to purified chondroitin sulfate A (Fried and Duffy, 1996) and also that placental malaria was often associated with the expression of the Var2CsA variant of PfEMP1, although other *var* genes were also transcribed at the same time (Duffy et al., 2006). In fact, the design of vaccine based upon fragments of Var2CsA has been attempted to inhibit placental colonization by *Plasmodium* and thus prevent congenital malaria (Fried and Duffy, 2015)

The severity of babesiosis ranges from mild, self-limited, febrile illness accompanied by nonspecific subjective symptoms to even life-threatening infection in immunocompromised individuals that is associated with severe hemolytic anemia, disseminated intravascular coagulation leading to acute respiratory distress syndrome, and even renal or hepatic failure. Hepatomegaly and splenomegaly are often observed in babesiosis patients, and a life-threatening complication of severe *B. microti* infection is spontaneous splenic rupture (Froberg et al., 2008; El Khoury et al., 2011; Farber et al., 2015; Dumic et al., 2018; Li et al., 2018; Patel et al., 2019) similar to fatal outcomes reported for malaria (Imbert et al., 2009; Chang et al., 2018; Elizalde-Torrent et al., 2018). Depending on the stage and severity of babesiosis, the marginal zone of spleen is eliminated, and white and red pulp regions merge. Since the red pulp region macrophages need to remove increased numbers of dead and infected RBCs, enlargement of the red pulp region is often observed. The elasticity of the splenic membrane plays a key role in patient survival as sometimes the organ can be up to five times its normal size, as is the case during malaria. Recently, Kho et al. described the accumulation of biomass due to medium to high CD71^+^ expressing reticulocytes as well as non-phagocytosed parasites, thus providing another shelter for the parasite’s survival and replication, contributing to splenomegaly (Kho et al., 2021). We also demonstrated the presence of free parasites in the spleens during the murine model of *B. microti* infection (Djokic et al., 2018a); however, neither RBC aggregations nor biomass accumulation was observed (Srivastava et al., 2015). Human architecture details for spleen involvement are scarce for babesiosis such that it is difficult to draw any conclusions. Transfusion transmitted babesiosis in already immunocompromised recipients with a variety of underlying medical conditions results in complicated disease such that death can occur in all age group patients (Herwaldt et al., 2011; Fang and McCullough, 2016; Gray and Herwaldt, 2019). Transmission of babesiosis can also occur through infected solid organ transplantation (Herwaldt et al., 2011; Gray and Herwaldt, 2019). Overall, severe babesiosis is more of a health problem in elderly, splenectomized, and immunocompromised individuals, while *Plasmodium* species, particularly *P. falciparum*, can cause brain hemorrhage and death even in immunocompetent young persons.

## Discussion

*Plasmodium* and *Babesia* are two parasites that belong to the class Apicomplexa and are erythrocyte-infecting protozoa. The life cycles of these two protozoa overlap greatly despite their difference as modes of transmission. *Plasmodium* spp. are transmitted by adult infected *Anopheles* female mosquitoes during their quick blood meals, while *Babesia*-transmitting ticks take days to imbibe blood during both the acquisition of this pathogen from one vertebrate host and during its transmission to another. Adult *Anopheles* female mosquitoes are able to acquire gametocytes from hosts, which then undergo sexual cycle, and then these mosquitoes transmit sporozoites during the next blood meal. In contrast, different developmental stages of hard ticks are responsible for acquisition and transmission of *Babesia* species because each stage takes blood meal only once. Thus, if the larvae of ticks acquire *Babesia* gametocytes during blood meal from the infected host, after sexual cycle completion, only nymphs that develop after molting will be able to transmit sporozoites to the next host during their blood meal. The sporozoites infection of hepatocytes that occurs during the *Plasmodium* asexual cycle in the host has not been reported to occur during the *Babesia* infection cycle. Since donated blood is not widely tested for *Babesia* presence, transfusion-transmitted babesiosis, and not malaria, remains a significant healthcare problem. Both parasites can also exhibit transplacental transmission from a pregnant mother to her child. The major differences in their asexual cycle are that *Plasmodium* species replicate in the PV inside the host cells surrounded by PVM in which PTEX insertion allows the secretion and uptake of molecules (Ho et al., 2018). This parasite also undergoes schizogony to produce a large number (~6-36/iRBC) of merozoites (Antinori et al., 2012). In contrast, *Babesia* species multiplies within the host erythrocyte cytoplasm, undergo only one to three replication cycles, and lack schizogony. Unlike *Plasmodium* species, *Babesia* is not susceptible to heme produced as a result of the degradation of hemoglobin during infection.

Innate immune response during the early stages of infection by malaria parasites in the liver is mediated by NK and NKT cells. At the same time, *Plasmodium* RNA is suggested to cause pro-inflammatory IFN-I stimulation through an interferon-α/β receptor-dependent manner that acts together with IFN-γ production (Liehl et al., 2014). This partnership results in phagocytosis-mediated killing by macrophages. The role of IFN-I is not explored yet for babesiosis likely because the liver stage is absent for this pathogen. Depletion of NK cells in mice did not impair the clearance of *B. microti* parasitemia, while macrophage depletion made mice susceptible to *B. rodhaini*, emphasizing the role of these phagocytes in the hemoparasites (Li et al., 2012). DCs produce pro-inflammatory cytokines and play a crucial role during the immunity and pathogenesis of the malaria parasite. They provide a link between innate and adaptive immune responses. For example, IL-12 produced by DCs activates NK cells to induce the production of IFNγ, which primes macrophages and neutrophils to enhance phagocytosis (Walsh and Mills, 2013; King and Lamb, 2015). Macrophages both act as antigen-presenting cells to induce adaptive immune response and promote Th1 and effector T cell responses. During the erythrocytic cycle, the host macrophages play a critical role in the resolution of parasitemia during both malaria and babesiosis, and together with cellular immunity also offer cross-protection against other related hemoparasites. In fact, *B. microti* in the susceptible mouse model of infection showed the importance of macrophages in the resolution of parasitemia (Djokic et al., 2018a; Djokic et al., 2018b; Djokic et al., 2019).

Both cellular and humoral immune responses are significantly induced during *Babesia* and *Plasmodium* infection. Antibodies block *Plasmodium* merozoite invasion of erythrocytes; however, they do not appear to significantly promote opsonophagocytosis of iRBCs of either *Babesia* or *Plasmodium* (Moreau et al., 2015; Arora et al., 2018; Akter et al., 2019; Kochayoo et al., 2019; Oyong et al., 2019; Efstratiou et al., 2020). Therefore, humoral immunity appears to play a minor role in resolving the parasitemia of these parasites. CD8+ cells are involved in the clearance of *Plasmodium* in hepatocytes by producing NO and IFNγ and also contributing immune cells during the *P. vivax* erythrocytic cycle (Doolan and Martinez-Alier, 2006; Chakravarty et al., 2008; Kelemen et al., 2019). CD4+ cells play the traditional helper cells role for cellular and humoral immune responses. Th1 response plays a critical role in the clearance of both protozoa and the resolution of both malaria and babesiosis. Inflammatory CD4+ T cells offer protection against malaria by producing IFNγ and TNFα such that *P. falciparum* infection or its replication is inhibited, thus preventing severe malaria (Boyle et al., 2015). Interestingly, this phenomenon is more prominent in adults than children (Boyle et al., 2017). On the other hand, IL-10 producing CD4+ cells show a correlation with parasite burden and likely regulate immune response such that immunopathology is restricted (Drewry and Harty, 2020). Similarly, pronounced inflammatory response to *B. duncani* WA1 strain infection results in mouse fatality due to excessive inflammatory cytokine, such as TNFα production (Hemmer et al., 2000), while IL-10 producing CD4+ cells play effector roles during *B. microti* infection in mice that show high parasitemia and anemia, but infection does not result in death (Djokic et al., 2018b; Djokic et al., 2019). The crucial roles of CD4+ cells during *B. microti* infection are also demonstrated experimentally since treatment of mice with anti-CD4 MAbs diminished protection against challenge infection. In addition, CD4 cell deficient mice failed to completely clear parasitemia, and the transfer of CD4 depleted spleen cells to naïve mice challenged with *B. microti* failed to resolve parasitemia efficiently (Igarashi et al., 1999; Skariah et al., 2017).

Both immunosuppression and immune evasion occur during infection with *Babesia*, and more prominently during *Plasmodium* infections (Fried and Duffy, 1996; Smith et al., 2000; Kassa et al., 2006; Nunes and Scherf, 2007; Fried and Duffy, 2017; Fried et al., 2017; Reiss et al., 2018; Djokic et al., 2019; Lee et al., 2020). Suppression of adaptive immune response by both of these parasites does not seem to affect their own clearance; however, it exacerbates diseases caused by bacteria that may coinfect the hosts with these protozoa. Both parasites result in splenomegaly and even hepatomegaly. Cytoadherence-mediated sequestration of iRBCs in different organs during malaria infection is a hallmark of immune evasion demonstrated by *P. falciparum*, while latent hypnozoites forms of *P. vivax* can survive hiding in the liver of patients for a long time and can result in the relapse of malaria. Cerebral malaria is the most serious consequence of *P. falciparum* infection and sequestration, while placental sequestration results in congenital malaria. Sequestration of *Babesia* species in organs has not been carefully investigated until now. Although the general symptoms of babesiosis are similar to malaria and congenital transmission has also been reported (Wormser et al., 2015; Saetre et al., 2018), the most serious disease-causing mortality due to *Babesia* infection occurs in immunocompromised, splenectomized, and elderly patients due to multi-organ failure. While severe malaria occurs even in immunocompetent humans of different ages, healthy individuals usually remain asymptomatic after *Babesia* infection.

**Figure 3:**
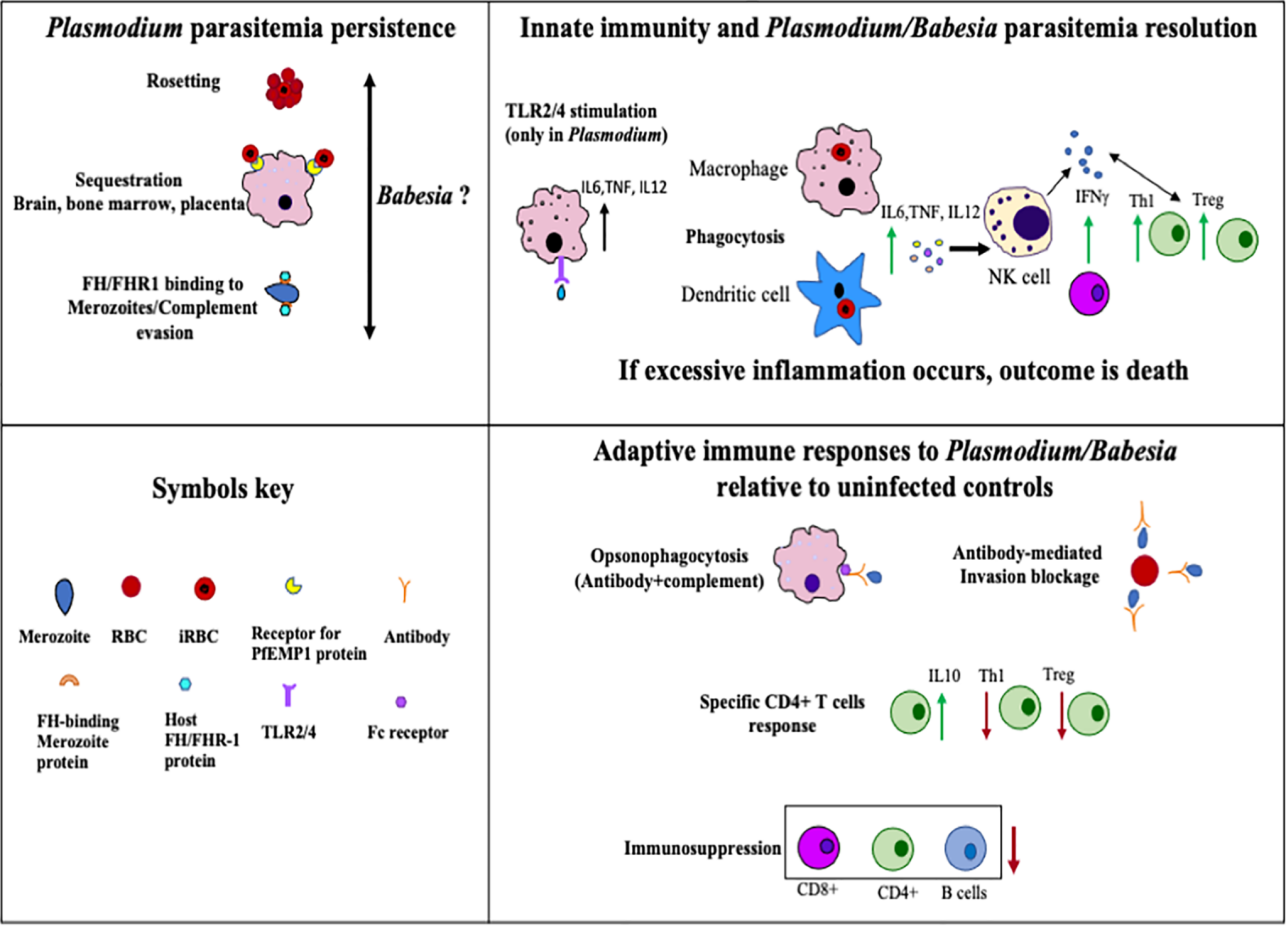
Summary of host immune responses stimulated during *Plasmodium* and *Babesia* species infection that help in the clearance of parasites, cause death of the host due to excessive inflammation, or allow protozoan survival in the host for long periods of time. Uninfected individuals or animals were included as controls for comparison in various previous studies that formed the foundation of this figure.

The summary of general immune responses extracted from studies on infection with different species of *Plasmodium* and *Babesia* individually is provided in **Figure 3**. This cartoon figure depicts the major mechanisms reported for clearance of parasites, excessive inflammation mediated killing of the host(s), and immunosuppression relative to uninfected individuals or animal controls, protozoan sequestration, and immune evasion that facilitate their long-term survival in their respective hosts. Overall, this review summarizes the similarities and differences between *Plasmodium* and *Babesia* species infection cycles, the immune responses they generate, the immune evasion mechanisms employed by *P. falciparum* and *P. vivax*, and the immunosuppression caused by these two *Plasmodium* species as well as by *B. microti.* The impact of suppression of primarily adaptive immunity by these parasites on coinfecting bacterial pathogens is also summarized. Generally, the immunosuppression caused by these parasites is only mild; however, the attenuation of adaptive immunity exacerbates disease manifestations by coinfecting bacterial pathogens, which become more persistent and result in more severe diseases (Nordstrand et al., 2007; Lundqvist et al., 2010; Blank et al., 2016a; Blank et al., 2016b; Djokic et al., 2019) irrespective of the pathogenic species involved. This relatively poorly examined healthcare problem will need more attention in the future because it could result in more fit pathogen evolution due to the selection of the bacterial mutants that could accumulate during coinfections.

## Author Contributions

VD and NP wrote the initial draft of this review. SR generated data for **Figure 2**, and all three authors edited and read the final version. NP also prepared figures and table. All authors contributed to the article and approved the submitted version.

## Funding

This publication is supported by the National Institutes of Health (R01 AI137425) grant to NP.

## Conflict of Interest

The authors declare that the research was conducted in the absence of any commercial or financial relationships that could be construed as a potential conflict of interest.

## Publisher’s Note

All claims expressed in this article are solely those of the authors and do not necessarily represent those of their affiliated organizations, or those of the publisher, the editors and the reviewers. Any product that may be evaluated in this article, or claim that may be made by its manufacturer, is not guaranteed or endorsed by the publisher.
